# Glycosylation of Hemagglutinin and Neuraminidase of Influenza A Virus as Signature for Ecological Spillover and Adaptation among Influenza Reservoirs

**DOI:** 10.3390/v10040183

**Published:** 2018-04-07

**Authors:** Paul Kim, Yo Han Jang, Soon Bin Kwon, Chung Min Lee, Gyoonhee Han, Baik Lin Seong

**Affiliations:** 1 Department of Biotechnology, College of Life Science and Biotechnology, Yonsei University, 50 Yonsei-ro, Seodaemun-gu, Seoul 03722, Korea; yhjh0323@yonsei.ac.kr (Y.H.J.); yunbin829@hanmail.net (S.B.K.); onlysmile79@naver.com (C.M.L.); 2 Vaccine Translational Research Center, Yonsei University, 50 Yonsei-ro, Seodaemun-gu, Seoul 03722, Korea; kimpaul225@naver.com; 3 Department of Integrated OMICS for Biomedical Science, College of World Class University, Yonsei University, 50 Yonsei-ro, Seodaemun-gu, Seoul 03722, Korea; 4 Biomedicine Pharmaceutical Group, CJ Healthcare R&D Center, CJ HealthCare, 811 Deokpyeong-ro, Majang-myeon, Icheon 17389, Korea

**Keywords:** influenza, glycosylation, hemagglutinin, neuraminidase, reverse zoonosis, evolutionary biology, ecology

## Abstract

Glycosylation of the hemagglutinin (HA) and neuraminidase (NA) of the influenza provides crucial means for immune evasion and viral fitness in a host population. However, the time-dependent dynamics of each glycosylation sites have not been addressed. We monitored the potential N-linked glycosylation (NLG) sites of over 10,000 HA and NA of H1N1 subtype isolated from human, avian, and swine species over the past century. The results show a shift in glycosylation sites as a hallmark of 1918 and 2009 pandemics, and also for the 1976 “abortive pandemic”. Co-segregation of particular glycosylation sites was identified as a characteristic of zoonotic transmission from animal reservoirs, and interestingly, of “reverse zoonosis” of human viruses into swine populations as well. After the 2009 pandemic, recent isolates accrued glycosylation at canonical sites in HA, reflecting gradual seasonal adaptation, and a novel glycosylation in NA as an independent signature for adaptation among humans. Structural predictions indicated a remarkably pleiotropic influence of glycans on multiple HA epitopes for immune evasion, without sacrificing the receptor binding of HA or the activity of NA. The results provided the rationale for establishing the ecological niche of influenza viruses among the reservoir and could be implemented for influenza surveillance and improving pandemic preparedness.

## 1. Introduction

Influenza viruses have taken a heavy toll on public health through annual epidemics and occasional pandemics. Seasonal outbreak causes 3–5 million cases of severe illness and 250,000–500,000 deaths worldwide every year [1]. Occasionally, a new strain of influenza virus is transmitted to humans from an animal reservoirs, and can give rise to an influenza pandemic with considerably higher morbidity and mortality than seasonal epidemics [2]. Influenza A virus has an eight-segmented RNA genome and high levels of variability in viral strains due to a high propensity for genetic mutations. Two viral surface proteins, hemagglutinin (HA) and neuraminidase (NA) undergo continuous genetic changes, which enables the virus to escape host immune responses [3]. Moreover, the HA proteins undergo post-translational modifications by adding oligosaccharides to the consensus N-X-S/T (where X is any amino acid except proline) glycosylation motif [4]. Glycosylation in HA is important for the folding and stability of the protein [5,6], and, in some cases, significantly affects receptor binding and cleavage of the precursor HA0 protein, influencing the virulence and antigenicity of the virus [7]. Furthermore, recent studies have suggested that glycosylation on the HA globular head domain physically shields the antigenic sites, preventing antibody recognition and leading to viral evasion from antibody-mediated neutralization [8,9,10]. These findings support the general hypothesis that the acquisition of glycosylation on HA renders the virus resistant to neutralizing antibodies, a molecular mechanism by which a newly emerged influenza strain, such as a pandemic virus, may evolve into a seasonal strain among the human population. For instance, although the 1918 H1N1 influenza virus had only one glycosylation site (104th residue in H1 numbering) in the HA head domain, variant strains emerged thereafter with acquired additional multiple glycosylation sites in the domain [11]. Notably, the 2009 H1N1 pandemic strain essentially showed a glycosylation pattern that was the same as that of the 1918 pandemic strain, carrying only one glycosylation site at the same position in HA [11,12,13]. These findings imply that the glycosylation of HA is one of the influencing factors associated with the emergence of H1N1 pandemics. Although it has long been known that the viruses gain glycosylation sites as a mean of counteracting host immunity [3], the dynamics of glycosylations in establishing the ecological niche of influenza viruses among susceptible hosts, considering the concerted role of HA and NA on viral infection cycle and immune evasion, remains largely unknown.

**Figure 1:**
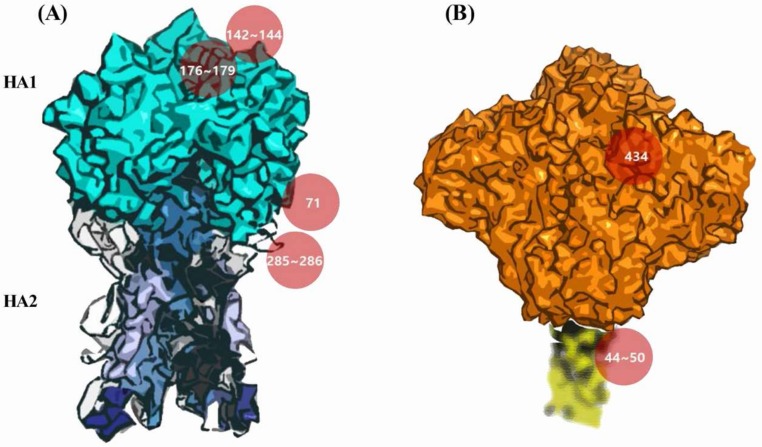
Structures of hemagglutinin (HA) and neuraminidase (NA) with major glycosylation sites. The HA structure (**A**) shows three clusters for pandemic relatedness (red). The positions 142 to 144 (cluster I) and 176 to 179 (cluster II) are located at the top of the HA1. The glycosylations at 285, 286 (cluster III), and 71 are identified on the side of HA. The NA structure (**B**) shows the switch in positions (44 to 50) located in the stem for pandemic status and an independent site (434 in the head domain) for seasonal adaptation (red). The Protein Data Bank (PDB) structure of the stem region (yellow dotted, blurred region) has not been determined yet. The complete absence of glycans in all three clusters of HA coupled with the switch in positions of NA (44 to 50) represent a hallmark of influenza pandemics. The cosegregation of glycosylation positions (HA 71 and NA 434), either the absence or presence at both positions, is identified as an independent characteristic of adaptation to humans and the reverse zoonosis into swine.

In this study, we analyzed and monitored the time-dependent glycosylation changes in HA and NA among influenza H1N1 viruses isolated during the past century. Through further analysis of previous in silico studies on the glycosylation of influenza antigens [14], we deduced a detailed mapping of glycan-masked epitopes (Figure 1) and identified potential pleiotropic effects on immune evasion. Although we confirmed that the emergence of H1N1 pandemics coincided with the sudden disappearance of particular glycosylation sites [11], our analyses newly found that the H1N1 swine influenza (A/New Jersey/76 H1N1) also had a glycosylation pattern remarkably similar to the 1918 and 2009 H1N1 pandemic strains. The short-lived 1976 H1N1 swine influenza was originally thought to have emerged in the Southern Hemisphere where it would have gained pandemic potential, but it was later concluded that the strain was restricted to spread in only Fort Dix [15,16,17]. There also have been particular glycosylation sites in NA that have been associated with influenza pandemics during the past century [14,18], although the biological role of NA glycosylation in viral pathogenesis and immune response has not been studied extensively to date [19]. The present analyses evidently showed that NA glycosylation was responsible for recent zoonotic transmission from swine [20,21]. Moreover, the current results suggest that glycosylation patterns serve as a predictive characteristic not only for influenza pandemics, but inter-species zoonotic and reverse zoonotic transmissions that should be closely monitored as part of the global surveillance efforts with respect to influenza viruses.

## 2. Materials and Methods

### 2.1. Prediction of Potential N-Glycosylation Sites in HA and NA of Influenza A H1N1 Viruses

Full-length amino acid sequences of H1N1 HA (5785 human, 224 avian, and 1448 swine isolates) and NA (2986 human, 210 avian, and 1764 swine isolates) were obtained from the Influenza Virus Resource (IVR) at the National Center for Biotechnology Information (NCBI) on April 2017. Only full-length sequences were subjected to analysis; partial or duplicated identical sequences were excluded. *N*-glycosylation sites were predicted using the NetNGlyc 1.0 of Center for Biological Sequence Analysis (CBS, Technical University of Denmark, Kemitorvet) to examine the sequence context of Asn-Xaa-Ser/Thr sequons [22]. NetNGlyc was chosen on the basis of its reported high prediction accuracy of 76% [22,23]. The program provides two measures of prediction confidence: (1) the average of prescore from nine artificial neural networks (2) and the “jury agreement” among the nine networks [22,23], where a score of >0.5 is taken as a threshold for occupation.

### 2.2. Phylogenetic Tree

Phylogenetic trees were constructed using Methods and Algorithms for Bioinformatics (MAB) phylogeny.fr analysis [24,25]. Amino acid sequences of the classical swine viruses from the the early 1930s, 1976, and the early 1990s [26] as well as human isolates were collected in FASTA format from NCBI. Each phylogenetic tree was obtained through the “one click” mode of phylogeny analysis using MUltiple Sequence Comparison by Log-Expectation (MUSCLE) for alignment, PhyML for phylogeny, and TreeDyn for drawing [24,25].

### 2.3. Homology Modeling, in Silico Protein Glycosylation, and Visualization

To determine and visualize the positions of glycosylation sites, we generated 3D structures of representative HA and NA proteins with different patterns of potential *N*-glycosylation sites in human influenza A (H1N1) viruses using SWISS-MODEL [27,28]. For adding specific glycosylation sites, the crystal structures of non-glycosylated pandemic strain A/California/04/2009 HA (PDB code: 3LZG) and NA (PDB code: 4B7Q) together were used as the model for human influenza H1N1. After homology modeling, glycans were added onto the potential *N*-glycosylation sites in HA and NA using the Glyprot Server [22,23]. All N-linked glycans (*N*-glycans) were selected as the basic conformation and the structures were visualized by the PyMOL Molecular Graphics System, version 1.8, Schrödinger, LLC (New York, NY, USA).

### 2.4. Structural Analysis

The accessible surface area of the HA and NA antigenic sites [29,30,31] was calculated using environment and virtual docking of atoms (ENVA), a program based on the Shrake–Rupley algorithm [32,33]. The accessible surface area of each amino acid residue was calculated by taking the ratio of the exposed surface area to the shielded surface area for each atom. AutoDock Vina was used for the receptor–ligand binding analysis [4]. The receptor-binding site of HA (PDB code: 3UBJ) was used to extract its sialic acid structure and designate the GRID area. The NA structure (PDB code: 4B7Q) was used to search for the active site.

## 3. Results

### 3.1. Dynamics of N-Linked Glycosylations in the HA and NA of Influenza H1N1 Viruses

**Figure 2:**
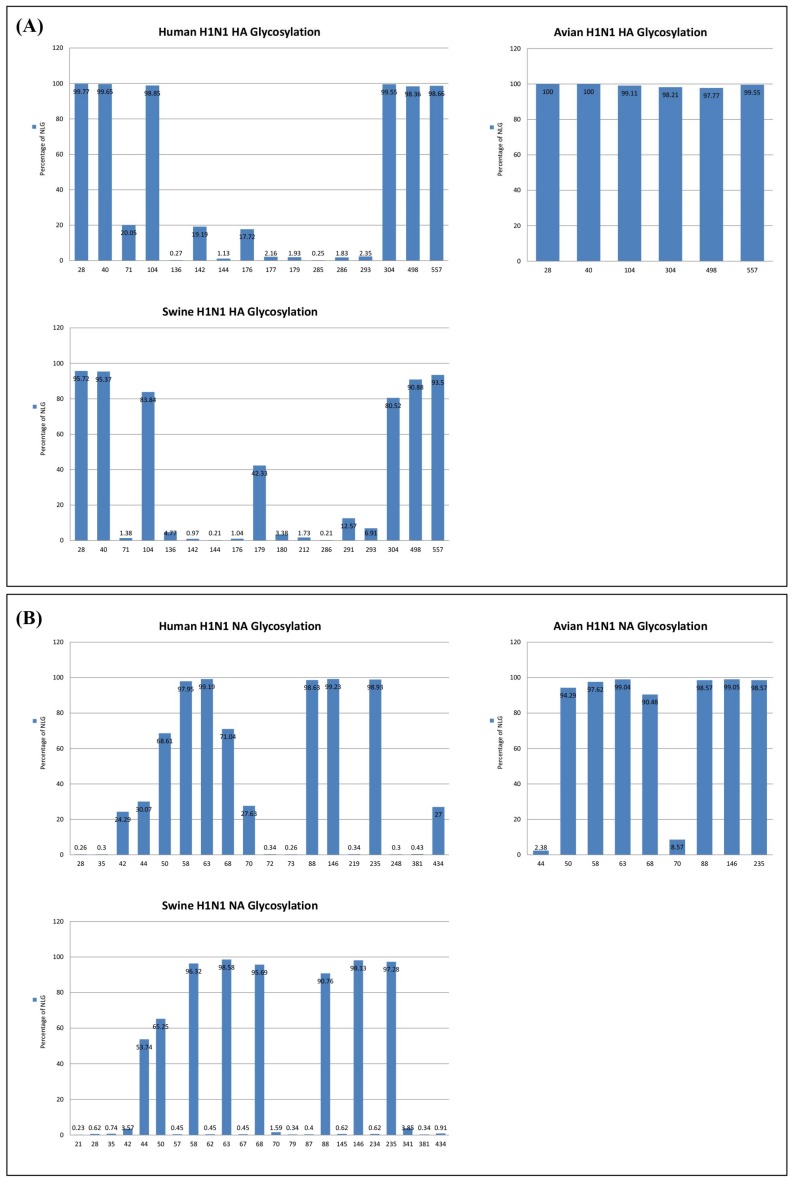
Total glycosylation sites predicted for H1N1 HA and NA. HA sequence analysis: 5785 HA sequences were isolated from humans, 224 from avian sources, and 1448 from swine sources (**A**). NA sequence analysis: 2986 sequences were from humans, 210 from avian sources, and 1764 sequences were from swine isolates (**B**).

**Figure 3:**
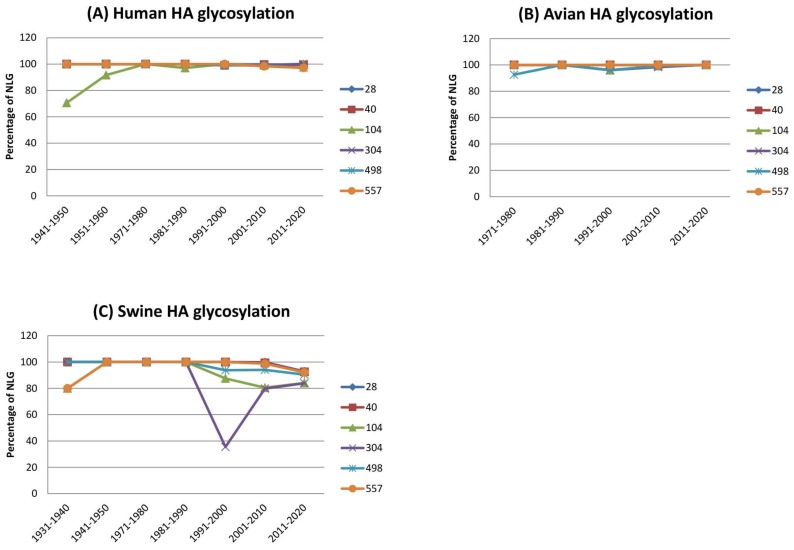
Conservation of N-linked glycosylation (NLG) sites of H1N1 HA per species. The six conserved HA NLG positions (28, 40, 104, 304, 498, and 557) of human (**A**), avian (**B**), and swine (**C**) isolates were analyzed for every decade from 1930 to present. The swine NLG position 304 was more variable than other sites.

Potential N-linked glycosylation (NLG) sites in the complete sequences of HA and NA proteins of influenza viruses isolated from avian, human, or swine species during the period 1918–2017 were predicted using NetNglyc 1.0 (Kemitorvet, Denmark). The analysis included 224 avian isolates, 5785 human isolates, and 1448 swine isolates. The overall contour structures of HA and NA are shown in Figure 1, with major glycosylation sites relevant for discussion on host adaptation throughout the manuscript. Avian isolates had a total of six NLG sites at positions 28, 40, 104, 304, 498, and 557, all of which were invariable over a ~40-year period among >97% of the total 224 viruses analyzed (Figure 2). These same six NLG sites were also highly conserved among >98% of the human isolates and >83% of the swine isolates (Figure 3). HA sequences from both human isolates and swine isolates harbored an additional 10 and 11 NLGs, respectively, with frequencies ranging from 0.27% to 20.05% and 0.21% to 42.33%, respectively (Figure 2). It is likely that the additional NLGs play important roles in the adaptation of avian influenza viruses and their potential for incorporation into human or swine hosts. Among the additional 10 NLG sites in the HA of human isolates, seven NLG sites could be grouped into three different clusters: 142–144 in cluster I, 176–179 in cluster II, and 285–286 in cluster III (Figure 1). Being located in very close proximities, none of the viruses harbored more than two NLGs within a single cluster; only one amino acid was glycosylated within a cluster in a virus without any exceptions (Figure S1).

The glycosylation of the NA also was analyzed among influenza H1N1 viruses from 2986 human isolates, 210 avian isolates, and 1764 swine isolates. The NA of avian influenza isolates carried nine glycosylation sites at positions 44, 50, 58, 63, 68, 70, 88, 146, and 235, seven of which were invariable in >90% of the sequences analyzed (Figure 2B). Among the seven invariable NLGs in avian influenza viruses, two NLGs (50 and 68) showed decreased frequencies of 68% and 71% in human strains, whereas the remaining five NLGs retained high frequencies of >97%. In swine influenza viruses, a single NLG at position 50 showed a decreased frequency of 65% compared with the same NLG in avian viruses, whereas the remaining six NLGs were highly conserved with frequencies of >90%. The NAs of human and swine influenza viruses acquired five additional NLGs in common at positions 28, 35, 42, 381, and 434 and harbored four (72, 73, 219, and 248) and nine (21, 57, 62, 67, 79, 87, 145, 234, and 341) unique NLGs, respectively. Our analysis revealed that most NLGs in the HA and NA of avian influenza viruses are invariable with high frequencies of >90% and that these NLGs also remain conserved in human and swine influenza viruses, suggesting that they play an essential role in viral survival and viability in hosts. Furthermore, it can also be speculated that the dynamic modulation of NLGs in HA and NA provides the molecular mechanisms by which avian influenza viruses cross the species barrier to infect humans or swine.

### 3.2. N-Linked Glycosylations as a Predictive Signature of Influenza Pandemics

**Figure 4:**
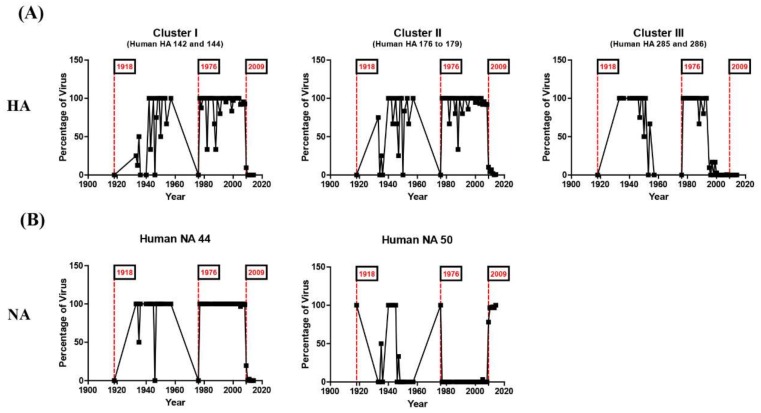
N-linked glycosylation patterns of HA and NA that have distinct differences among pandemic and seasonal H1N1 strains. The glycosylation patterns of HA in (**A**) and NA in (**B**) for human isolates between 1918 and 2015 were analyzed. No glycosylation was observed in all three glycosylation clusters in the 1918 and 2009 pandemics as well as in the 1976 “abortive” pandemic [34], which later turned out to be a local outbreak [15,16]. A gradual increase in glycosylations in cluster II was observed in swine HA in recent years, except for the year 1939, when glycosylations occurred in all three clusters. The NA glycosylations at positions 44 and 50 are mutually exclusive. In human isolates, glycosylation at position 44 was not present for all three (1918, 1976, and 2009) pandemics.

**Figure 5:**
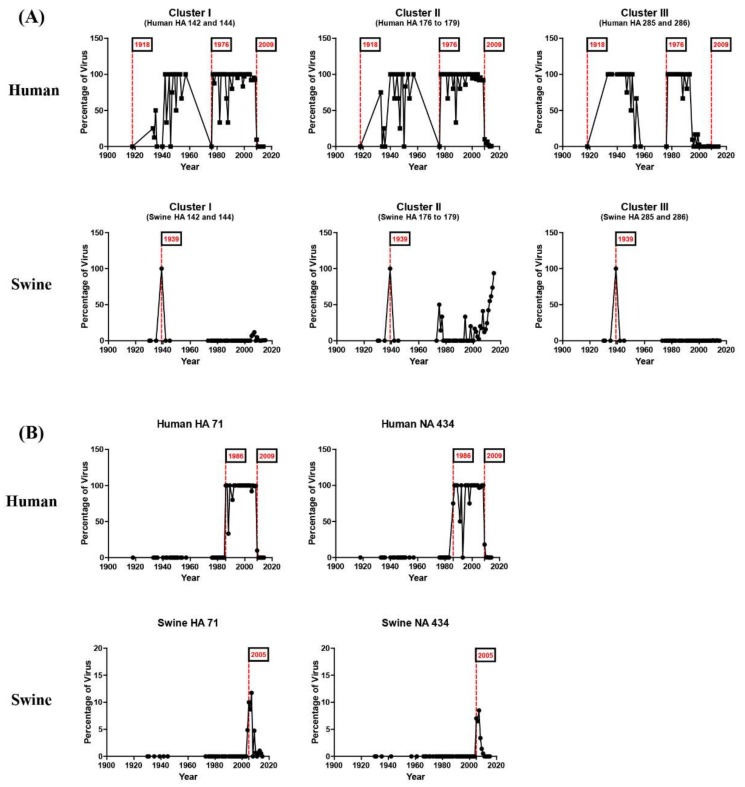
The reverse zoonosis of influenza predicted by glycosylation patterns of H1N1 HA and NA. The glycosylation patterns of HA and NA for human and swine species isolated between 1918 and 2015 were analyzed. A gradual increase in glycosylations in cluster II was observed in swine HA in recent years, except for the year 1939 when glycosylation occurred in all three clusters (**A**). The position 71 glycosylation in HA and the position 434 glycosylation in NA were simultaneously identified from 1986 to 2009 in human isolates (**B**). The cosegregation of the two independent mutations could be considered as a genetic characteristic of adaptation to humans. The swine isolates had a glycosylation at position 71 in HA and position 434 in NA in 2005, indicating “reverse zoonosis” from humans into swine.

Time-dependent changes in the NLG frequency of each cluster showed an interesting and meaningful pattern. In the case of time-dependent analysis, we separated the HA and NA sequence as before and after 2015 to identify novel recent glycosylation patterns. NLGs were absent from all three of the clusters in the HA of the 1918 pandemic strains but increased to high frequencies in human isolates that emerged after the pandemic (Figure 4). Again, the NLGs in the same region completely disappeared in the 1976 virus and were fully restored in isolates from the following year, 1977. More notably, clusters I and II were completely devoid of NLGs in the HA of the 2009 pandemic strains. Thus, NLGs in clusters I and II have shared very similar variations throughout the past century, including their sudden disappearances in pandemic viruses and their rapid and full restoration following the pandemic. Interestingly, the disappearance of NLGs in cluster III appears to have preceded that in clusters I and II (Figure 4). Strikingly, our analysis showed that the typical NLG sites in all clusters were empty in the 1918 and 2009 H1N1 pandemics as well as in the 1976 viruses (Figure 4). In 1976, swine influenza A/New Jersey/76 (H1N1) caused human infections among recruits at Fort Dix, infecting more than 200 soldiers and resulting in 13 severe cases and one death. This incidence prompted an immediate vaccination of >40 million people in the USA, ceasing further transmission [15,16,34] and culminating in an “abortive pandemic” [34]. It has been speculated that the 1918 pandemic and the 2009 pandemic were caused by the sudden introduction of a novel virus into humans from animals, such as birds and pigs [35,36]. Our analysis provides further evidence for such a hypothesis. Considering that swine and avian influenza viruses rarely carry NLGs in the three clusters described (Figure 5, Figure S2), it is likely that all three pandemic events were caused by interspecies viral transmission from birds or pigs to humans [35,37]. The variations of the NLGs in the NA of human influenza H1N1 viruses also yielded pandemic-associated patterns. Glycosylation at position 44 was completely absent in the 1918 and 2009 pandemics and, interestingly enough, in the 1976 abortive pandemic as well (Figure 4). In contrast, glycosylation at position 50 was present in all three pandemics. Thus, a marked shift in the glycosylation site from 44 to 50 in NA could serve as a characteristic of a pandemic. Remarkably, it was not only the HA protein displaying the same lack of glycosylation in all three clusters from all three pandemics during the past century, but also the NA protein. Together, these findings expound on a previous report [18] and newly identify the 1976 virus as carrying the same genetic predisposition for pandemic status.

### 3.3. Reverse Zoonosis of Influenza Viruses Is Traceable by N-Linked Glycosylation

Comparisons of NLGs at the same positions between human and swine strains demonstrated an interesting pattern. In the HA of swine influenza viruses, NLGs have been virtually absent in all three clusters throughout the past century (Figure 5). The only exception was the year 1939 when a sudden appearance of NLGs in all three of the clusters was noted; however, it immediately disappeared the following year (Figure 5). Considering that the three NLG clusters are found only in the HA of human strains, this distinctive pattern strongly implies that the HA genes from the 1939 swine viruses were introduced from human viruses rather than by genetic drift of classical swine viruses. Indeed, it was reported that the 1939 swine virus infections caused symptoms similar to human influenza, with coughing, sneezing, and pneumonia in pigs, and all such genes were shown to be more closely related to human strains than to classical swine strains [38]. Further expounding on previous studies, our data suggest that the 1939 swine viruses had glycosylation clusters that were the same as those of human viruses, suggesting a “reverse zoonosis” of human-to-swine transmission. Phylogenetic analysis supports our prediction. The HA of the 1939 swine influenza virus was more closely related to that of previous human influenza viruses than to classical swine viruses (Figure S3).

**Figure 6:**
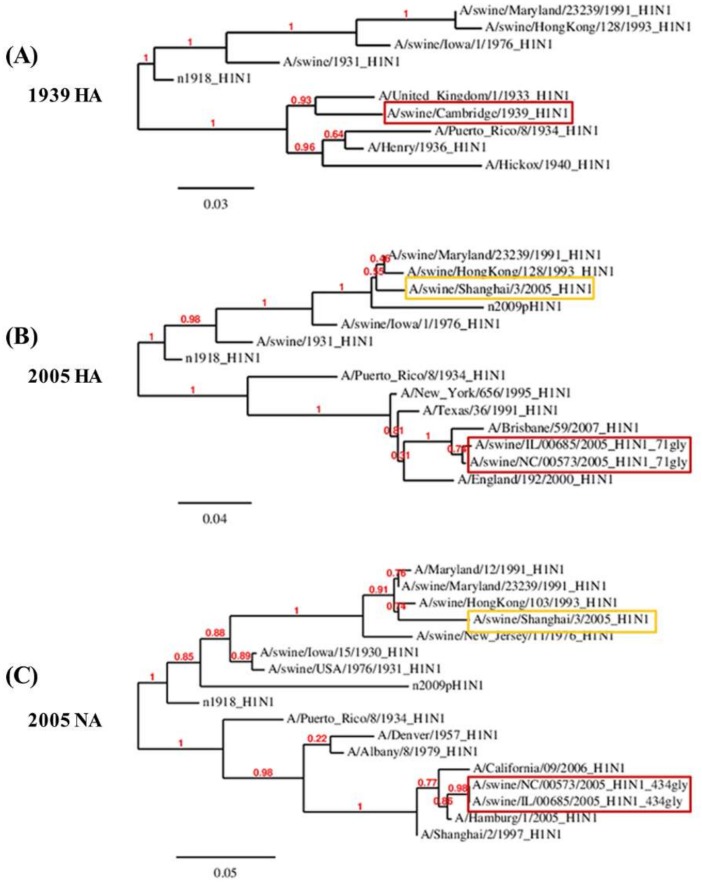
Phylogenetic tree of specific swine isolates that have incorporated human glycosylation patterns. A phylogenetic tree was used for analyzing the sequence correlation of swine isolates carrying glycosylation patterns of human isolates (rectangular red) among other classical swine and human seasonal flu isolates. The 1939 swine HA was very similar to a 1933 human influenza HA isolate (**A**). The 71 glycosylated HA in (**B**) and 434 glycosylated NA in (**C**) from 2005 swine isolates matched human isolates; however, the non-glycosylated 2005 swine isolates at HA 71 and NA 434 (rectangular yellow) had low similarities with human isolates.

NLGs at HA 71 and NA 434 positions between human strains and swine strains also provide evidence for reverse zoonosis. The HA 71 and NA 434 NLGs appeared almost simultaneously in human influenza viruses (Figure 5). These occurred most likely by genetic mutations rather than interspecies transmission from pigs or birds, given that no NLGs were present prior to that time in the two positions in human, swine, or avian influenza viruses (Figure 5; Figure S2). These two NLGs first appeared in 1986 among human strains and subsequently among swine strains between 2005 and 2009 with frequencies of 5–10%, indicating cocirculation of viruses with and without NLGs. Phylogenetic analysis shows that the HA of the swine viruses with NLGs are antigenically closer to human strains than to classical swine strains, whereas the swine viruses without NLGs were classical swine strains (Figure 6; Figure S3). This result suggests that at least the HA genes of the 2005–2009 swine influenza viruses originated from human influenza viruses, as independent evidence of the reverse zoonosis of human-to-swine transmission.

### 3.4. N-Linked Glycosylation in Human Influenza H1N1 Viruses Emerged after the 2009 Pandemic

**Figure 7:**
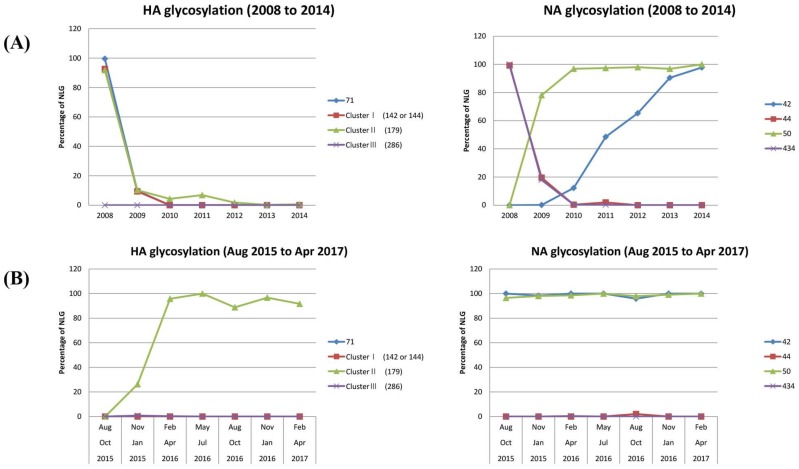
The temporal changes in glycosylation sites of H1N1 HA and NA among recent isolates. Major glycosylation sites were analyzed among influenza isolates from 2008 to 2014 in (**A**) and recently (**B**) (from August 2015 to April 2017): three clusters in HA and positions 44/50 in NA as signatures for pandemics and position 71 in HA and positions 42/434 in NA as characteristics for zoonotic (swine to human) transmission. Conserved glycosylation sites on HA and NA that are not related to pandemic or zoonotic transmissions are not shown.

After the 1976 abortive pandemic, the NLGs in the three clusters of the HA and NLG-44 of the NA were fully restored the following year (Figure 4). After the 2009 pandemic, however, the HA remained completely devoid of NLGs in the clusters until early 2016 when position 179 (within cluster II) became glycosylated (Figure 7). It is interesting to note that the HA of swine H1N1 viruses has been glycosylated in position 179 in recent years (Figure 5), and the human H1N1 HA appears to have followed the same (Figure 7). In the case of NA, although NLGs at positions 44 and 50 have followed previous patterns, as shown in Figure 4, NLG at position 42 has emerged for the first time during this century (Figure 7). As shown in Figure S4, NLG-42 in NA first appeared in swine isolates at the beginning of the new millennium and then human isolates began to accumulate the same glycosylation in 2014, probably reflecting a zoonotic transmission from swine to human. The novel glycosylation of this position appears to be gaining momentum in recent years within all three isolates (human, avian, and swine) and coexists with glycosylation at position 50 (Figure 7).

### 3.5. Potential Antigenic Shielding by N-Linked Glycosylation

It has been widely acknowledged that NLGs can shield antigenic epitopes in the HA of influenza viruses, a method by which viral variants escape antibody neutralization induced by prior exposures to earlier strains [8,12,13,39]. Our analysis shows that seasonal influenza viruses that emerge after a pandemic rapidly accumulate multiple NLGs in the HA until the next pandemic (Figure 4), supporting the theory of antigenic shielding by *N*-glycans. It is reasonable to assume from this data that the most primary function of NLGs in HA is to promote viral immune evasion that allows successful adaptation into seasonal strains. To address this issue, we used in silico and determined if the addition of an NLG could shield the antigenic epitopes of HA. Using the HA of A/Korea/1/09 (H1N1) virus as a template, four NLGs were introduced individually into the three clusters plus position 71 to generate _142_NHTV, _177_NLSK, _286_NVTV, and _71_NCTV mutants. First, we calculated changes in the accessible surface area and polarity of each amino acid residue in five antigenic epitopes: Ca, Cb, Sa, Sb, and H1C. Our calculation suggested that, among the five epitopes, Sa, Sb, and H1C were significantly affected by NLG-142 in cluster I, NLG-177 in cluster II, or NLG-286 in cluster III (Table 1). In contrast, the Ca and Cb epitopes were not appreciably affected. Thus, in silico prediction showed that the acquisition of an NLG at cluster I (position 142) or cluster II (position 177) physically shielded the antigenic site Sa by decreasing the accessible surface area. This is not surprising because both NLGs are positioned within the antigenic site Sa (comprising positions 141–142 and 170–181). Likewise, NLG-286 (in cluster III) resulted in the direct shielding of the antigenic site H1C. Remarkably, NLG-177 (cluster II) markedly shielded other antigenic sites as well, in particular, positions 202, 206, and 212 of the site Sb, abrogating the accessible surface area of multiple amino acid residues and changing their polarity (Table 1). Such a pleiotropic effect of a single NLG on multiple epitopes of Sa and Sb was not anticipated. NLG-71 did not have an appreciable effect on the HA antigenic sites. These results suggest that NLGs at the major glycosylation hotspots would affect the antigenicity and interfere with antibodies binding to the three major epitopes in HA, thus contributing to immune escape by the virus. Our predictions are favorably consistent with previous experimental observations, wherein mutant viruses were generated by reverse genetics, and the introduction of NLGs at positions 142 and 177, but not 71, significantly influenced the antigenicity and virulence of the 2009 pandemic H1N1 [39].

The accessible surface areas (exposed surface) and polarities are shown as percentages. A significant effect by glycosylation is marked in red whenever appropriate. The wild-type A/Kor/1/09(H1N1) amino acid sequence is shown in the second column. In HA, glycosylation at position 142 affected residues 141 and 142 in Sa and glycosylation of 177 affected residues 177, 202, 206, and 212 in Sb. Glycosylation at 286 decreased the accessible surface area and polarity of 286 in H1C. Glycosylation of position 212 affected the residues 202, 206, 211, and 212 in Sb. In NA, glycosylation at 434 and 341 appeared to affect AS5, but the effect was not as significant as in HA.

**Figure 8:**
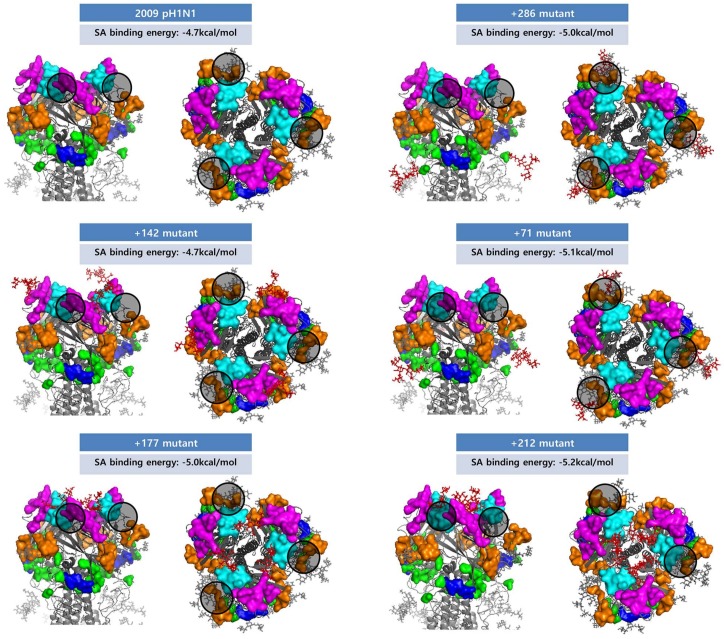
Glycosylated H1 trimer structures and sialic acid binding affinities. The H1 antigenic sites Ca, Cb, Sa, Sb, and H1C are shown in orange, blue, magenta, cyan, and green, respectively. The glycans newly introduced by mutations are marked in red. The predicted sialic acid binding affinities are shown, and the binding sites are represented with black circles. The 142, 177, and 212 glycosylations are located on top of the HA head domain, whereas the 286 and 71 glycosylations are on the side of the domain. The structures are represented as viewed from the side (**left**) and top (**right**).

In our prediction, the potential NLG sequon 212 NNTY of the A/Pavia/65/2016(H1N1), one of the human isolates from the year 2016, was newly identified (Figure S5). Prior to 2016, NLG-212 had been found in swine isolates only (Figure 2A), and A/Pavia/65/2016(H1N1) was reported as a swine origin virus causing a severe acute respiratory distress syndrome in a middle-aged man [40]. Although the NLG score was 0.4749 under the threshold of 0.5, where the potential for glycosylation was operationally defined as negative (Figure S5), we assumed that the glycosylation patterns of swine isolates could be introduced into human H1N1 viruses. The swine-specific glycosylation sites comprise eight positions (156, 157, 160, 212, 239, 275, 423, and 425) in HA and seven positions (104, 123, 171, 186, 341, 362, and 416) in NA (Figure 2). Except for HA position 212 and NA position 341, most glycosylations were found in only one or two cases (Table S1). To address the potential effect of the glycosylation on epitopes, the structures of the pH1N1 mutant HA (212 ADAY to NHTY as the mutated sequence with the highest glycosylation score in NetNglyc) and NA (341 NGAN to NGTN) were generated. NLG-212 had an antigenic shielding effect on AS Sb in the structure-based prediction, and it may have affected the antigenicity and antibody binding in HA as a function of the three NLG clusters. We compared the structure and properties of NLG-434 (NLG of seasonal H1N1 NA) and NLG-341 (swine-specific NLG) by introducing them to the pH1N1 NA structure; however, they had no significant effects on antigenic sites (Table 1). To examine the potential effects of NLGs on receptor binding by HA, we set up a grid box at an HA–sialic acid binding site and simulated the docking process using AutoDock Vina (version 4) [4]. The 3D structures of HA binding to sialic acid were previously reported for A/Puerto Rico/8/34(H1N1) and A/California/04/2009(H1N1) [41,42]. For each HA structure, binding models were extracted, and the binding affinity between HA and sialic acid was calculated. No appreciable differences were observed among wild-type HA and glycosylation mutants, with affinity values ranging from −5.2 to −4.7 kcal/mol (Figure 8). In the case of NA, introducing potential glycosylations on pH1N1 did not affect the sialic acid binding affinity. These data suggest that glycosylation patterns are exploited by influenza viruses as a means to escape host immunity without sacrificing the viral fitness for causing infection.

## 4. Discussion

Our present study revealed that NLGs in the HA and NA of influenza H1N1 viruses are intimately involved in the ecology and evolution of the viruses. The NLG frequencies in the HA and NA of avian, human, and swine influenza viruses isolated over nearly a century showed that the acquisition of additional NLGs in the HA and NA are required to cross species barriers into human or swine hosts. By tracking NLG variation patterns in the HA and NA of human influenza viruses on a yearly basis, we found that specific NLGs synchronized with influenza pandemic outbreaks and their evolution into seasonal influenza stains. In addition, a comparison of NLGs among the three species traced interspecific transmission events via zoonosis and reverse zoonosis. The glycosylation hotspots comprising cluster I (142–144), cluster II (176–179), and cluster III (285–286) simultaneously disappeared in the two H1N1 pandemics of 1918 and 2009. Of note, a similar absence of glycosylations was newly identified for the 1976 H1N1 viruses, mapped with the “abortive” pandemic [18,34]. The 1976 H1N1 swine influenza was originally thought to have emerged in the Southern Hemisphere where it would have gained pandemic potential, but turned out to be short-lived, restricting the infection only within Fort Dix [15,16,17]. The HA amino acid sequences of all three pandemic viruses were very similar with a 90% homology, and the antiserum from the 1976 swine flu-infected patients has been shown to neutralize the 2009 pandemic strain [17,34,43,44]. However, the 1976 and 1918 viruses showed differences in multiple residues in the H1 head domain (4, 11, 54, 62, 68, 90, 146, 154, 170, 173, 202, 217, 228, 239, 270, 272, 285, and 300) (Figure S6). Likewise, the H1 head domain of the 1976 virus also differed from that of the 2009 virus in multiple positions (10, 14, 62, 86, 88, 90, 172, 159, 200, 203, 233, 239, 241, 256, 275, 277, 287, and 295), suggesting that the origin of the 1976 swine flu was independent of the 1918 and 2009 pandemic viruses (Figure S6). Despite the differences, it is remarkable that the three viruses shared common glycosylation patterns completely lacking all three glycosylation hotspots. These results confirm and further expound on previous works suggesting that glycosylation on HA is associated with H1N1 pandemics [12].

**Figure 9:**
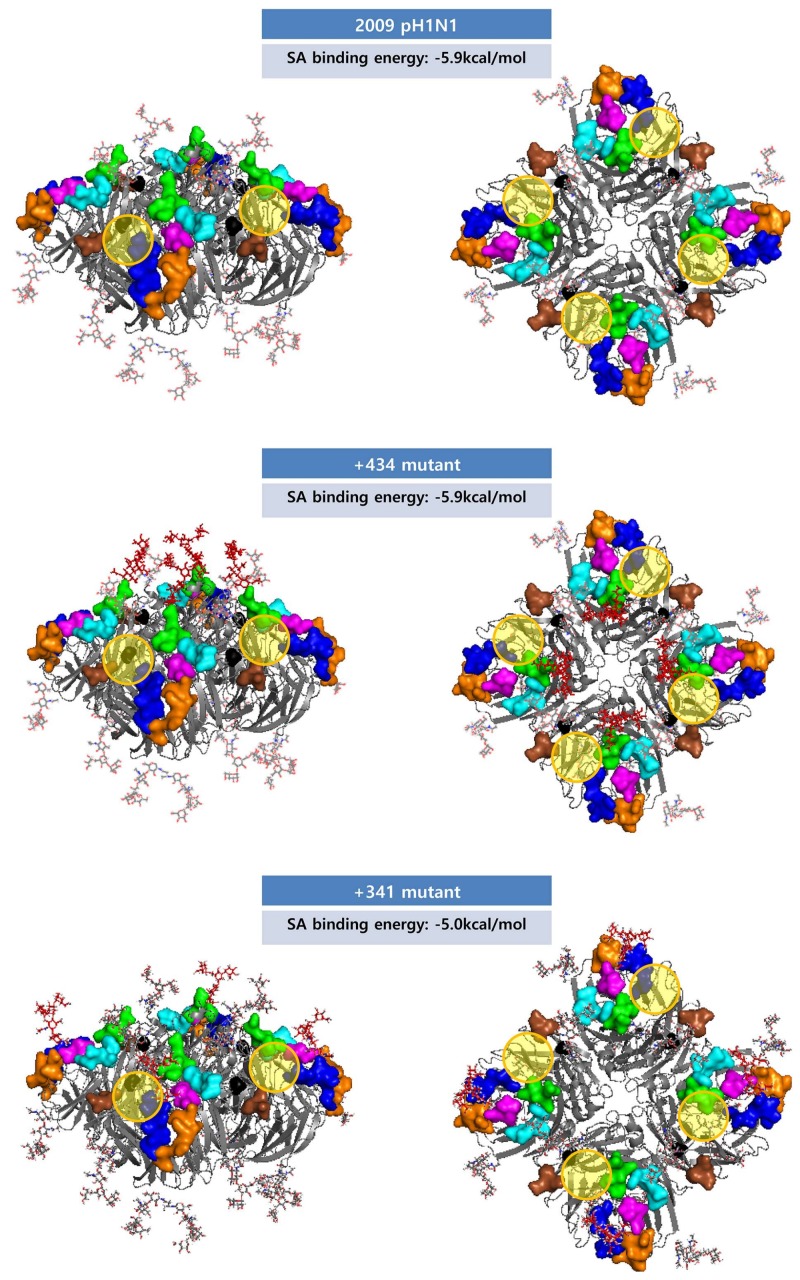
Glycosylated NA tetramer structures and the sialic acid binding affinities. The NA antigenic sites AS1, AS2, AS3, AS4, AS5, AS6, and AS7 are shown in orange, blue, magenta, cyan, green, brown, and black, respectively. The newly added glycans at positions 434 and 341 are marked in red. The predicted sialic acid binding affinities are shown for the NA tetramer structure. The binding sites are represented by yellow circles.

The present structure-based in silico prediction study further suggests that none of the major glycosylations in HA or NA (Figure 1) appear to affect the viral fitness throughout the infection cycle. Notably, any of the three “cluster” areas in HA do not significantly affect the binding of the cellular receptor sialic acid at the inception of infection (Figure 8). Likewise, the glycosylation in NA failed to affect sialic acid binding to the active site (Figure 9), without hampering the release of mature virions from the infected cells at the later stage of infection. It is remarkable, therefore, without affecting viral fitness, *N*-glycans at each cluster I, II, and III can independently shield the antigenic sites Sa, Sb, and H1C, for immune evasion (Table 1). Previously, it was shown that glycan addition generally impairs HA function, while dramatically increasing the viral fitness in the presence of a neutralizing environment [45]. Notably, a recent report shows that glycosylation facilitates immune evasion by compensating for the fitness cost of antigenic escape mutations [46]. In line with a powerful means for immune evasion without sacrificing viral fitness, the single 177 *N*-glycan caused a pleiotropic effect on both Sa and Sb affecting the exposed areas and polarities simultaneously (Table 1). Most of the seasonal influenza viruses are glycosylated at these positions (Figure 4), suggesting an interference with the binding of neutralizing antibodies, enabling viral escape from a pre-existing immunity. Changes in glycosylation patterns also were monitored in swine and avian isolates from the past century. The 1939 swine influenza was a unique event, particularly because of the high mortality and human-like symptoms, and, remarkably, it was glycosylated at all three HA hotspot sites simultaneously (Figure 5). The present analyses provide convincing evidence for a “reverse zoonosis” of human viruses into the swine population in 1939 [47,48].

The NA protein is also associated with glycosylation-dependent historical events of influenza outbreaks. First, glycosylation at positions 44 and 50 was mutually exclusive. Second, the shift from 44 to 50 earmarked the emergence of all three pandemics: 1918, 2009, including the “abortive” 1976 [34]. Third, for the last three decades, the swine H1N1 virus has been glycosylated at NA 44 with a gradual loss of glycosylation at NA 50 (Figure 4; Figure S7). Whether these changes will impact the future emergence of pandemics in humans remains a grave potential concern, particularly considering the role of swine as a “mixing vessel” for the generation of human viruses [49]. In addition, the HA 71 and NA 434 glycosylations were introduced into human isolates, remarkably at the same time over the past century (Figure 5), and the virus carrying these glycosylations was noted for its infectivity of both humans and pigs in 2005 [50]. Thus, cosegregation of the two independent mutations could be considered as a genetic characteristic of adaptation to humans. This cosegregation behavior perhaps is better explained by the opposing roles played by HA and NA during an influenza infection: HA facilitates entry into cells for the initiation of infection and NA facilitates the egress from infected cells [51]. Although the opposing roles could be counterbalanced by glycosylation on both surface antigens, the immunological mechanism for the cooperative behavior towards adaptation to humans requires further study. According to the Centers for Disease Control and Prevention reports, influenza activity in the USA began to increase steadily in late December 2015, and the influenza A virus has remained predominant [52]. Our own monitoring showed that the most recent H1N1 virus in 2016 gained full glycosylation at position 179 (cluster II; Figure 7), which potentially contributed to its increased activity in 2016 by allowing the virus to escape from pre-existing immunity developed during the 2009 pandemic period. Moreover, a novel NA 42 glycosylation has increased recently in prevalence (Figure S4). Since the 2009 pandemic, swine flu re-emerged in India in 2015 [20], and the virus acquired glycosylation at position 42 (Figure 7). NA 42 glycosylation was initially identified in swine isolates in the late 1990s and has been introduced to avian and human viruses in recent years (Figure S4), mostly likely by genetic reassortment. In retrospect, the 2009 pH1N1 was a triple reassortant of human, swine, and avian origins, where the HA and NA originated from swine influenza [49]. In light of the recent introduction of cluster II (179) glycosylation into the human H1N1 (Figure 7), additional glycosylations in cluster I (142 to 144), cluster II (285 to 286), or the swine-originated glycosylation patterns are expected to gain momentum in the foreseeable future, resulting in a gradual shift into seasonal adaptation. Independently, as discussed above, the cosegregation of specific glycosylations on HA 71 and NA 434 among human isolates suggests a cooperative behavior between the two surface antigens in zoonotic transmission that leads to successful adaptation in humans. Simultaneous changes in glycosylation patterns, such as their complete absence in HA and a marked shift in glycosylation sites in NA, should be taken as a characteristic predicting future pandemic outbreaks. The cooperative, cosegregational, and counterbalanced glycosylation on HA and NA, reflecting the opposite roles of the two surface antigens in an infection cycle [51], must operate to heighten viral fitness within the immune environment established in the human host. It should be emphasized, however, that the “signature” glycosylation pattern herein identified cannot on its own serve as an indicator of pandemics or spillover among different hosts. Other factors, e.g., viral internal genes other than HA and NA, and environmental effects that have an impact on influenza reservoirs and the host susceptibility to infection should also be taken into account. Eventually, the prognostic value of the present analyses should be subjected to experimental confirmation via well-established reverse genetic approaches [53,54]. We suggest a close monitoring of the HA and NA characteristic sites of the H1N1 virus to better predict future pandemics and seasonal adaptations. The mechanistic roles of glycosylation as a viral strategy for zoonotic transmission and human adaptation require further investigation; however, the immune correlates associated with glycosylation patterns of HA and NA will be useful for improving pandemic and seasonal influenza vaccines.

## Figures and Tables

**Table 1:** Accessible surface areas and polarities in the hemaggluninin (HA) and neuraminidase (NA) antigenic sites of H1N1 pandemic viruses and mutants.

(A) HA Glycosylation							
+142	Antigenic Site	Position	WT AA	WT acc (%)	Mutant acc (%)	WT pol (%)	Mutant pol (%)
	Sa	141	P	91	78	58	47
	Sa	142	N	73	33	69	17
+177	Antigenic Site	Position	WT AA	WT acc (%)	Mutant acc (%)	WT pol (%)	Mutant pol (%)
	Sa	177	K	67	16	87	43
	Sb	201	T	45	39	83	69
	Sb	202	S	68	3	82	33
	Sb	206	Q	54	25	83	59
	Sb	212	A	58	0	83	24
+286	Antigenic Site	Position	WT AA	WT acc (%)	Mutant acc (%)	WT pol (%)	Mutant pol (%)
	H1C	286	D	76	32	85	47
+71	Antigenic Site	Position	WT AA	WT acc (%)	Mutant acc (%)	WT pol (%)	Mutant pol (%)
	-	-	-	-	-	-	-
+212	Antigenic Site	Position	WT AA	WT acc (%)	Mutant acc (%)	WT pol (%)	Mutant Pol (%)
	Sb	202	S	68	22	82	36
	Sb	206	Q	54	37	83	71
	Sb	211	N	38	1	70	33
	Sb	212	A	58	8	83	36
(B) NA Glycosylation							
+434	Antigenic Site	Position	WT AA	WT acc (%)	Mutant acc (%)	WT pol (%)	Mutant pol (%)
	AS5	429	G	0	0	43	56
+341	Antigenic Site	Position	WT AA	WT acc (%)	Mutant acc (%)	WT pol (%)	Mutant pol (%)
	AS5	430	T	37	25	73	66

## Supplementary Materials

The following are available online at http://www.mdpi.com/1999-4915/10/4/183/s1, Table S1: List of swine-only glycosylations in H1N1 HA and NA, Figure S1: Glycosylation “hotspot” clusters among human H1N1 hemagglutinin (HA) sequences, Figure S2: The variation of glycosylation sites of HA and neuraminidase (NA) in avian H1N1 isolates, Figure S3: Phylogenetic tree of specific swine isolates incorporating human glycosylation patterns (more detailed), Figure S4: Glycosylation in NA position 42 from recent human, swine, and avian isolates, Figure S5: A comparison of potential N-glycosylation sites from human isolates in Pavia between 2015 and 2016, Figure S6: Differences in the amino acid sequences of HA among 1918, 1976, and 2009 H1N1 viruses and Figure S7: Variation in the glycosylation of NA positions 44 and 50 of H1N1 swine isolates.
