# Supplementary material for: Glycosylation of Hemagglutinin and Neuraminidase of Influenza A Virus as Signature for Ecological Spillover and Adaptation among Influenza Reservoirs
Source: Viruses. 2018 Apr 7;10(4):183. doi: 10.3390/v10040183 (PMC5923477; doi:10.3390/v10040183)
Supplement: Supplementary file 1 [file viruses-10-00183-s001.pdf]

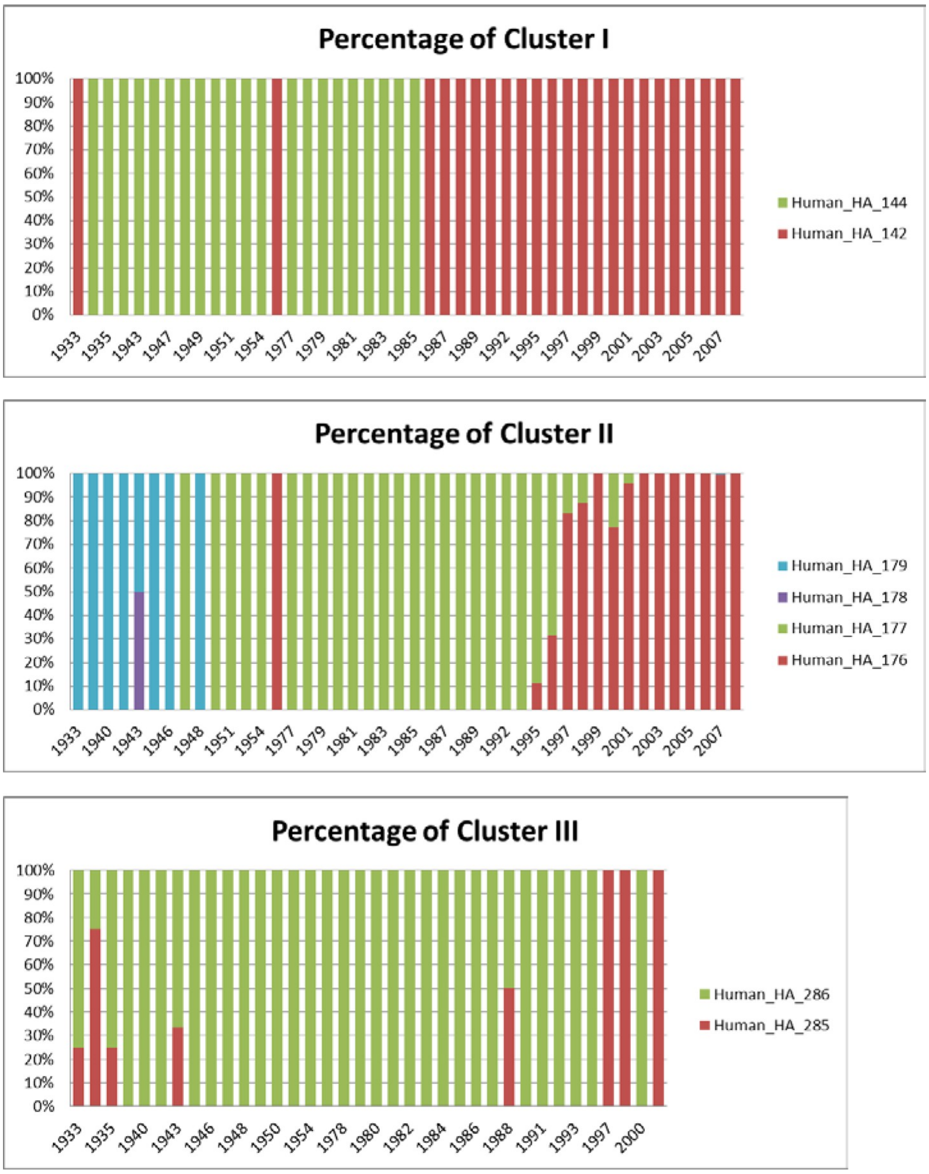


**Figure S1.** Glycosylation "hotspot" clusters among human H1N1 hemagglutinin (HA) sequences.

The probability of glycosylation for each hotspot cluster was calculated by the sum of the proportions of each amino acid constituting the clusters. The well-integrated time of each cluster is shown.

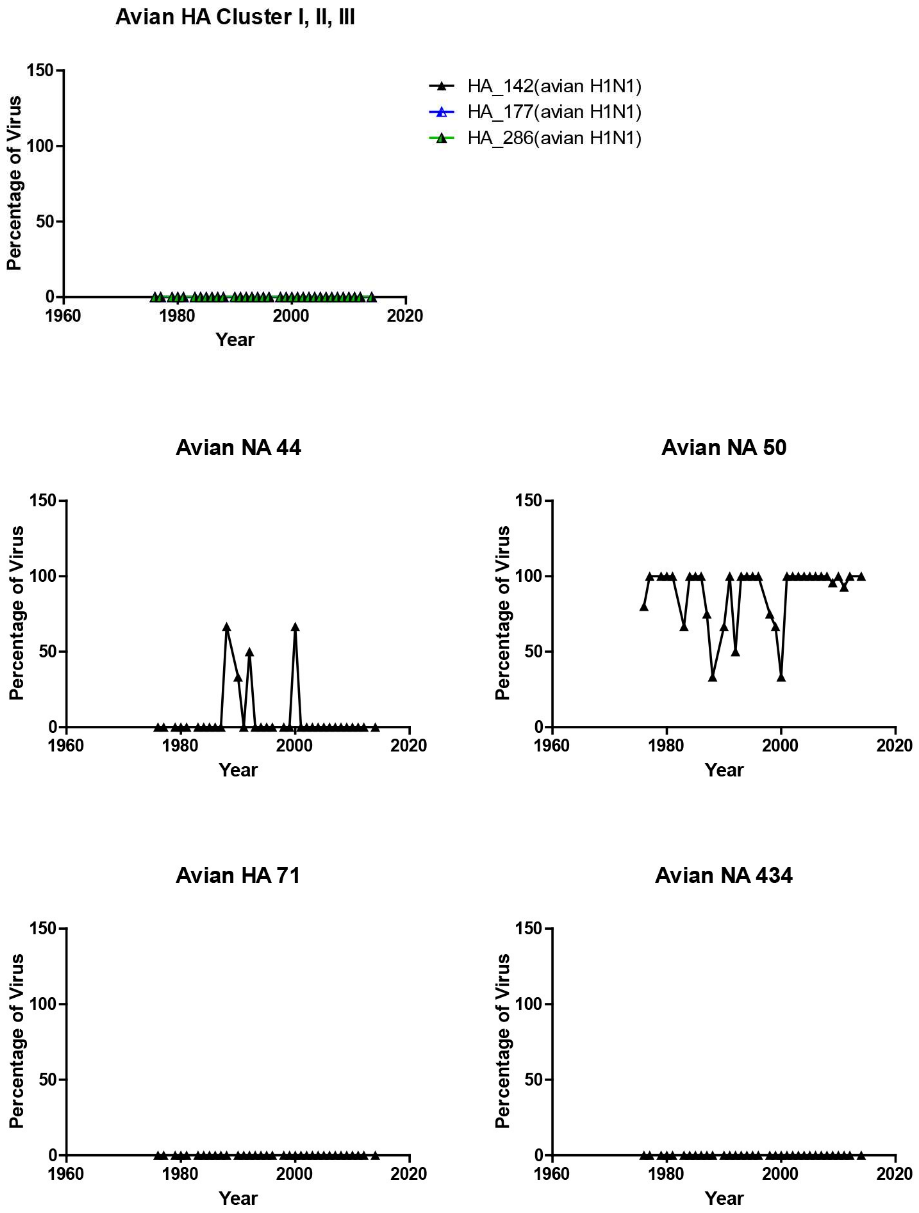


**Figure S2.** The variation of glycosylation sites of HA and neuraminidase (NA) in avian H1N1 isolates.

The avian H1N1 HA showed no glycosylation at any of the significant positions, such as pandemic-associated positions (142HA, 177HA, 286HA, 44NA, and 50NA) and reverse zoonosis-related positions (71HA and 434NA). The NA glycosylations at positions 44 and 50 are mutually exclusive. The majority of avian isolates show glycosylation at position 50.

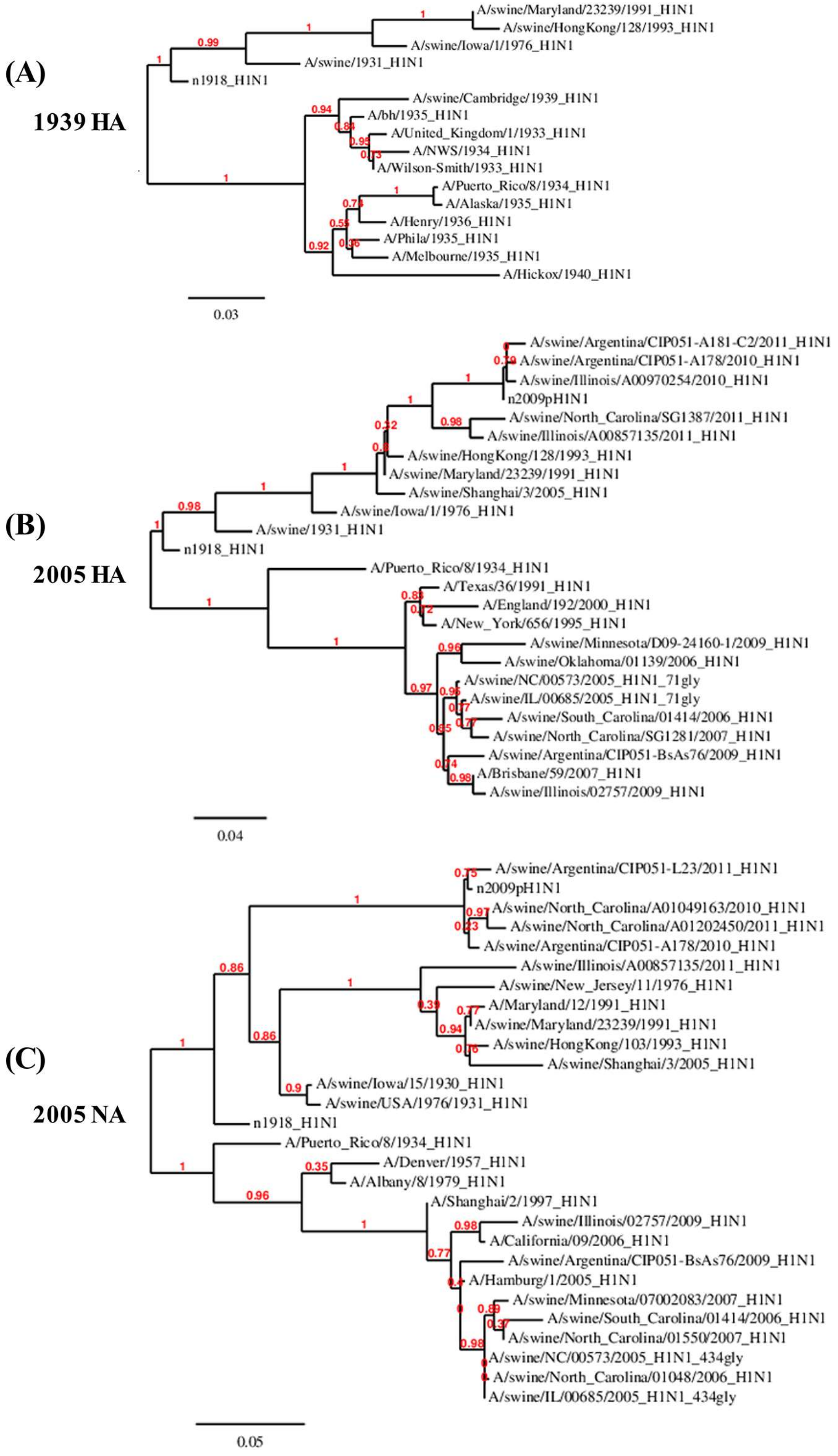


**Figure S3.** Phylogenetic tree of specific swine isolates incorporating human glycosylation patterns (more detailed).

A phylogenetic tree was used to analyze the sequence correlation of swine isolates carrying glycosylation

patterns from human isolates among other classical swine flu isolates and human seasonal flu isolates. The 1939 swine HA was very similar to 1930's human isolates. The 71 glycosylated HA and 434 glycosylated NA of swine isolates from 2005 to 2009 matched human isolates from 2005; however, the non- glycosylated swine HA and NA had low similarities with human isolates.

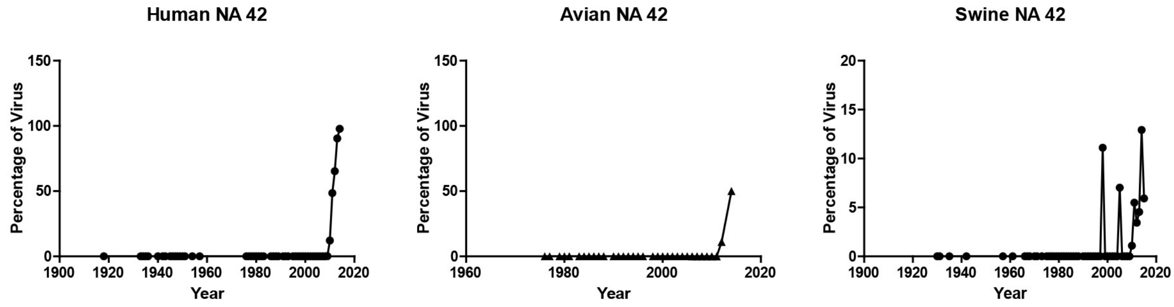


**Figure S4.** Glycosylation in NA position 42 from recent human, swine, and avian isolates.

The NA 42 glycosylation has been dormant for the last century but has begun to increase over the past few years.

**A/Pavia/VR10504/2015(H1N1)**

(Threshold=0.5)

| SeqName | Position | Potential | Jury agreement | N-Glyc result |
|---|---|---|---|---|
| ALM06089.1 | 27 NNST | 0.3900 | (8/9) | - |
| ALM06089.1 | 28 NSTD | 0.7996 | (9/9) | +++ |
| ALM06089.1 | 40 NVTV | 0.7476 | (9/9) | ++ |
| ALM06089.1 | 104 NGTC | 0.6429 | (8/9) | + |
| ALM06089.1 | 293 NTTC | 0.4864 | (5/9) | - |
| ALM06089.1 | 304 NTSL | 0.6710 | (9/9) | ++ |
| ALM06089.1 | 498 NGTY | 0.5208 | (4/9) | + |
| ALM06089.1 | 557 NGSL | 0.6834 | (9/9) | ++ |

**A/Pavia/65/2016(H1N1)**

(Threshold=0.5)

| SeqName | Position | Potential | Jury agreement | N-Glyc result |
|---|---|---|---|---|
| APT36456 | 27 NNST | 0.4144 | (7/9) | - |
| APT36456 | 28 NSTD | 0.7960 | (9/9) | +++ |
| APT36456 | 40 NVTV | 0.7511 | (9/9) | +++ |
| APT36456 | 104 NGTC | 0.4901 | (3/9) | - |
| APT36456 | 212 NNTY | 0.4749 | (6/9) | - |
| APT36456 | 291 NCTT | 0.4489 | (4/9) | - |
| APT36456 | 498 NGTY | 0.6293 | (9/9) | ++ |
| APT36456 | 557 NGSL | 0.6830 | (9/9) | ++ |

**Figure S5.** A comparison of potential N-glycosylation sites from human isolates in Pavia between 2015 and 2016. The introduction of potential glycosylation site 212 on influenza H1N1 human isolate is shown. The H1N1 human isolates did not possess an N-glycosylation sequon on position 212 until 2015. In 2016, the A/Pavia/65/2016(H1N1) had the 212 sequon as NNTY; however, the potential score was 0.4749, and the N-glycosylation result was operationally defined as negative.

| Position | 2 | 4 | 6 | 10 | 11 | 14 | 15 | 21 | 53 | 62 | 64 | 68 | 78 | 85 | 86 | 88 | 90 | 101 | 103 | 114 |
|---|---|---|---|---|---|---|---|---|---|---|---|---|---|---|---|---|---|---|---|---|
| 1918 | E | R | L | C | A | A | T | I | S | K | I | Q | L | D | L | L | A | N | E | D |
| 2009 | K | I | L | Y | T | T | A | L | R | G | V | H | I | E | S | D | A | S | D | D |
| 1976 | K | I | V | C | T | A | T | L | K | R | I | H | L | E | L | L | V | N | D | N |

| Position | 130 | 146 | 145 | 146 | 147 | 154 | 155 | 159 | 163 | 166 | 169 | 172 | 173 | 183 | 185 | 196 | 200 | 202 | 203 | 217 |
|---|---|---|---|---|---|---|---|---|---|---|---|---|---|---|---|---|---|---|---|---|
| 1918 | K | E | T | T | K | S | Y | S | K | L | T | G | S | V | N | V | P | G | T | S |
| 2009 | R | D | S | N | K | P | H | K | K | I | V | G | N | I | D | I | S | S | A | F |
| 1976 | R | E | T | N | R | P | Y | N | R | I | V | E | N | V | N | I | P | S | T | F |

| Position | 222 | 224 | 225 | 226 | 228 | 233 | 239 | 241 | 251 | 256 | 266 | 267 | 269 | 274 | 275 | 277 | 278 | 284 | 287 | 295 |
|---|---|---|---|---|---|---|---|---|---|---|---|---|---|---|---|---|---|---|---|---|
| 1918 | K | N | R | R | T | A | D | A | L | T | I | A | W | L | N | G | S | T | A | K |
| 2009 | R | S | K | K | K | I | D | E | V | K | V | V | R | M | E | N | A | I | T | T |
| 1976 | K | N | R | K | K | A | G | A | I | T | V | V | R | M | N | G | S | I | A | K |

| Position | 300 | 305 | 315 | 319 | 325 | 331 | 338 | 362 | 382 | 383 | 391 | 416 | 419 | 454 | 460 | 467 | 471 | 490 | 492 | 497 |
|---|---|---|---|---|---|---|---|---|---|---|---|---|---|---|---|---|---|---|---|---|
| 1918 | H | S | V | E | R | M | I | I | Q | K | G | N | R | F | R | K | K | N | A | R |
| 2009 | K | T | I | K | K | L | V | V | L | K | E | H | K | Y | K | R | K | D | T | K |
| 1976 | K | T | V | E | K | M | I | I | Q | R | G | H | K | F | K | R | R | D | T | K |

**Figure S6.** Differences in the amino acid sequences of HA among 1918, 1976, and 2009 H1N1 viruses.

The sequence homology identified by BLAST search. The homology between 1976 and 1918 and that between 1976 and 2009 were high (~90%), but the positions were different. Yellow: different positions between 1976 and 1918. Purple: different positions between 1976 and 2009.

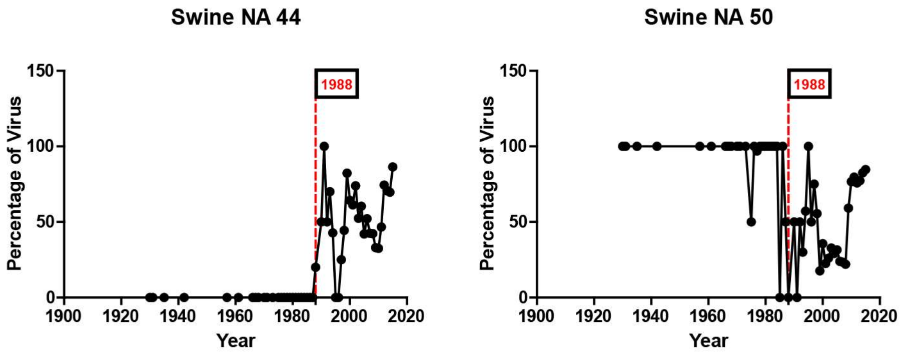


**Figure S7.** Variation in the glycosylation of NA positions 44 and 50 of H1N1 swine isolates.

The NA positions 44 and 50 are mutually exclusive, and the glycosylation at position 44 has been gradually increasing in swine isolates.

**Table S1.** List of swine-only glycosylations in H1N1 HA and NA.

| (A) Swine HA Glycosylation | | (B) Swine NA Glycosylation | |
|---|---|---|---|
| Position | Number of Isolates | Position | Number of Isolates |
| 156 | 1 | 21 | 4 |
| 157 | 1 | 104 | 1 |
| 160 | 1 | 123 | 2 |
| 202 | 1 | 186 | 1 |
| 204 | 3 | 212 | 2 |
| 211 | 2 | **341** | **68** |
| **212** | **25** | 362 | 1 |
| 239 | 2 | 416 | 2 |
| 240 | 1 | | |
| 275 | 4 | | |
| 423 | 1 | | |
| 425 | 1 | | |

A total of 1448 HA and 1764 NA sequences from swine isolates are analyzed. The number of isolates that have specific glycosylations is shown. The glycosylations of HA 212 and NA 341, in particular, were pronounced (bold).
